# Thanking *Cell & Bioscience* reviewers

**DOI:** 10.1186/2045-3701-3-3

**Published:** 2013-01-11

**Authors:** Yun-Bo Shi

**Affiliations:** 1 Editor-in-Chief, Cell & Bioscience

## Abstract

**Contributing reviewers:**

The editors of *Cell & Bioscience* would like to thank all the reviewers who contributed to the journal in Volume 2 (2012).

## 

Christopher Allen

United States of America

Jeff Barrow

United States of America

Esteban Celis

United States of America

Yonglong Chen

China

Taiping Chen

United States of America

Lin-Feng Chen

United States of America

Kaifan Dai

United States of America

Donald DeFranco

United States of America

Xiaoli Du

United States of America

Xin-Hua Feng

United States of America

Weichao Guo

United States of America

Yingbo He

United States of America

Xi He

United States of America

Kun Huang

United States of America

Harald Huber

Germany

Anders Jacobsson

Sweden

Hassan Jijakli

Belgium

Dong-Yan Jin

Hong Kong

Hung-Ying Kao

United States of America

Sander Kersten

Netherlands

Atif Khan

United States of America

Therese Kinsella

Ireland

Ah-Ng Tony Kong

United States of America

Ajit Kumar

United States of America

Ning Lei

United States of America

Kangguang Lin

United States of America

Yusen Liu

United States of America

Hui-Wen Lo

United States of America

Jianfeng Lu

United States of America

JIanjie Ma

United States of America

Vladimir Majerciak

United States of America

Franz Oswald

Germany

Bala Ramaswami

United States of America

Rachel Reinhard

Germany

Guangwen Ren

United States of America

Thomas Scanlan

United States of America

Sven Schinner

Germany

Changshun Shao

United States of America

Rajesh Sharma

United States of America

Yufang Shi

China

Songtao Shi

United States of America

Michal Stachowiak

United States of America

Hsin-Hsiung Tai

United States of America

Hiroshi Takeshima

Japan

Tse-Hua Tan

Taiwan

Moon-Shong Tang

United States of America

Walter Wahli

Switzerland

Zhong Wang

United States of America

Xin Wei Wang

United States of America

Shiliang Wang

United States of America

Weidong Wang

United States of America

Luan Wen

United States of America

Jiemin Wong

China

T.-C. Wu

United States of America

Yuntao Wu

United States of America

Hui Xiao

China

Jing Yan

United States of America

Yingzi Yang

United States of America

Paul Yen

Singapore

David Yu

United States of America

Ying Zhang

United States of America

Xiaoren Zhang

China

Bin Zhang

United States of America

Zhen Zhang

United States of America

Hui Zhao

Hong Kong

Zhongming Zhao

United States of America

Zhi-Ming Zheng

United States of America

